# The Chicken Egg Genotoxicity Assay (CEGA): Assessing Target Tissue Exposure and Metabolism in the Embryo‐Fetal Chicken Livers

**DOI:** 10.1002/em.70015

**Published:** 2025-05-12

**Authors:** Yax Thakkar, T. Kobets, Anne Marie Api, J. D. Duan, G. M. Williams

**Affiliations:** ^1^ Research Institute for Fragrance Materials, Inc. Mahwah New Jersey USA; ^2^ Department of Pathology, Microbiology and Immunology New York Medical College Valhalla New York USA

**Keywords:** chicken egg model, genotoxicity, liver, metabolism, new approach methodology, target tissue exposure

## Abstract

The Chicken Egg Genotoxicity Assay (CEGA) is an avian egg‐based model that utilizes the livers of developing chicken embryo‐fetuses to assess the ability of chemicals to produce direct DNA damage. The main goal of the study was to evaluate target tissue exposure and metabolism in the CEGA to assess its suitability as a biologically relevant new approach methodology (NAM) for detecting the genotoxic potential of chemicals. An imaging study using two‐photon excitation microscopy after the administration of a fluorescent dye (acridine orange) verified that chemicals following administration into the air sac of the fertilized chicken egg reach the target organ, liver. A metabolism study using liquid chromatography with high resolution mass spectrometry (LC/MS), conducted after the administration of benzo(a)pyrene (B(a)P) according to the CEGA protocol, confirmed the formation of sufficient amounts of reactive metabolite(s) responsible for the genotoxic effects of a parent compound upon reaching the target tissue. Moreover, an RNA sequencing study revealed that B(a)P in embryo‐fetal chicken livers significantly upregulated several genes responsible for the activity of the CYP1A1 enzyme, which is critical for the bioactivation of B(a)P. These findings, along with the previously reported DNA damage (i.e., DNA adducts and single‐strand breaks) produced by B(a)P in CEGA, support sufficient target tissue exposure to B(a)P and the ability of avian fetal livers to bioactivate B(a)P to a reactive intermediate. Overall, the findings in the study support the conclusion that the CEGA can be considered a robust potential alternative to the animal testing strategy for assessing the genotoxic potential of chemicals.

## 1 Introduction

Carcinogenic chemicals can be broadly classified based on their mode of action, with a major focus on genotoxic and non‐genotoxic mechanisms (Kobets et al. 2019). Genotoxic carcinogens interact with the genetic material of cells, causing mutations, chromosomal fragmentation, or rearrangements. These alterations can disrupt normal cellular functions, leading to uncontrolled cell proliferation and ultimately cancer.

Genotoxicity of chemicals can be evaluated in various in vitro and in vivo assays, which are mainly designed to evaluate potential to cause gene mutation, chromosomal damage, and DNA damage/repair pathways interruption (Kirkland et al. 2011). Due to the recent restrictions in the use of in vivo genotoxicity assays (European Parliament and Council 2003), there is a need for biologically relevant new approach methodologies (NAMs) to be used as animal alternatives for evaluating the genotoxic potential of chemicals that had in vitro positive results.

Genotoxicity can be induced by direct DNA activity of the parent compound and/or its metabolite. As such, metabolism plays a crucial role in the bioactivation of many chemicals. This process is often required for the formation of reactive electrophilic intermediates that can then directly react with DNA (Kobets et al. 2019). Bioactivation of different classes of chemicals may differ, and produced metabolites may interact with different sites on macromolecules, including DNA.

Since many in vitro systems lack an intrinsic ability to metabolize chemicals, the induced rat liver S9 fraction is used as an exogenous metabolic activation system (Ames et al. 1973; Paolini and Cantelli‐Forti 1997). However, this exogenous source of metabolic enzymes may not include those that are important for phase II detoxification at sufficient levels to generate biologically relevant responses. Hence, current in vitro testing systems generate a high number of misleading outcomes in testing and prediction of carcinogens (Kirkland et al. 2007).

The Chicken and related Turkey Egg Genotoxicity Assays (CEGA and TEGA, respectively) (Williams et al. 2014; Iatropoulos et al. 2017; Kobets, Duan, et al. 2018; Kobets et al. 2016; Kobets, Iatropoulos, et al. 2018) were developed as metabolically competent (Kobets, Iatropoulos, et al. 2018; Perrone et al. 2004) NAMs for genotoxicity screening to potentially replace short‐term in vivo studies required for human safety assessment. CEGA uses fertilized, specific pathogen‐free eggs from the white leghorn chicken of undetermined sex. Since the termination of the embryos in CEGA is conducted on incubation day 11, at least 10 days before hatching, discomfort to the organism is precluded, as the nervous system of the embryos is not completely developed (Hughes 1953). Thus, in compliance with the Animals (Scientific Procedures) Act 1986, 2012, CEGA is not considered to be an animal model.

CEGA evaluates two endpoints, formation of DNA adducts by means of the ^32^P‐nucleotide postlabeling (NPL) assay (Phillips and Arlt 2014; Randerath et al. 1981; Reddy and Randerath 1986) and induction of DNA single‐strand breaks using the alkaline single‐cell gel electrophoresis (comet) assay (Brendler‐Schwaab et al. 2005; OECD 2016; Tice et al. 2000). Both techniques are widely used for the evaluation of chemical‐induced DNA damage (Himmelstein et al. 2009) produced by either direct or indirect mechanisms; thus, elucidation of the mode of action of chemical carcinogens is possible. Additionally, fetal avian livers express the majority of the phase‐I and phase‐II biotransformation enzymes. Such metabolic capabilities allow the model to detect DNA‐reactive chemicals that only induce DNA damage after metabolic transformation (Kobets, Iatropoulos, et al. 2018; Perrone et al. 2004; Rifkind et al. 1979). Moreover, CEGA can efficiently detoxify and eliminate chemicals similar to the in vivo systems.

To establish the validity of a test system, it is critical to demonstrate target tissue exposure. This is especially valuable in cases when the system is used as a follow‐up for chemicals that produced adverse outcomes in the in vitro mammalian cell‐based assays (ICH S2(R1) 2011). The evidence of target tissue exposure can be demonstrated either by demonstrating cytotoxicity to the target tissue or by directly measuring a drug or related toxic metabolite in the target tissue. In vivo, assessment of cytotoxicity can be conducted by histopathological evaluation of the target tissue or by analyzing changes in the blood biochemistry values. The direct measurement of drug‐related substances can be performed in blood, plasma, or target tissues. Autoradiographic techniques can be used to assess tissue exposure to these substances (ICH S2(R1) 2011; Kirkland et al. 2019).

**FIGURE 1:**
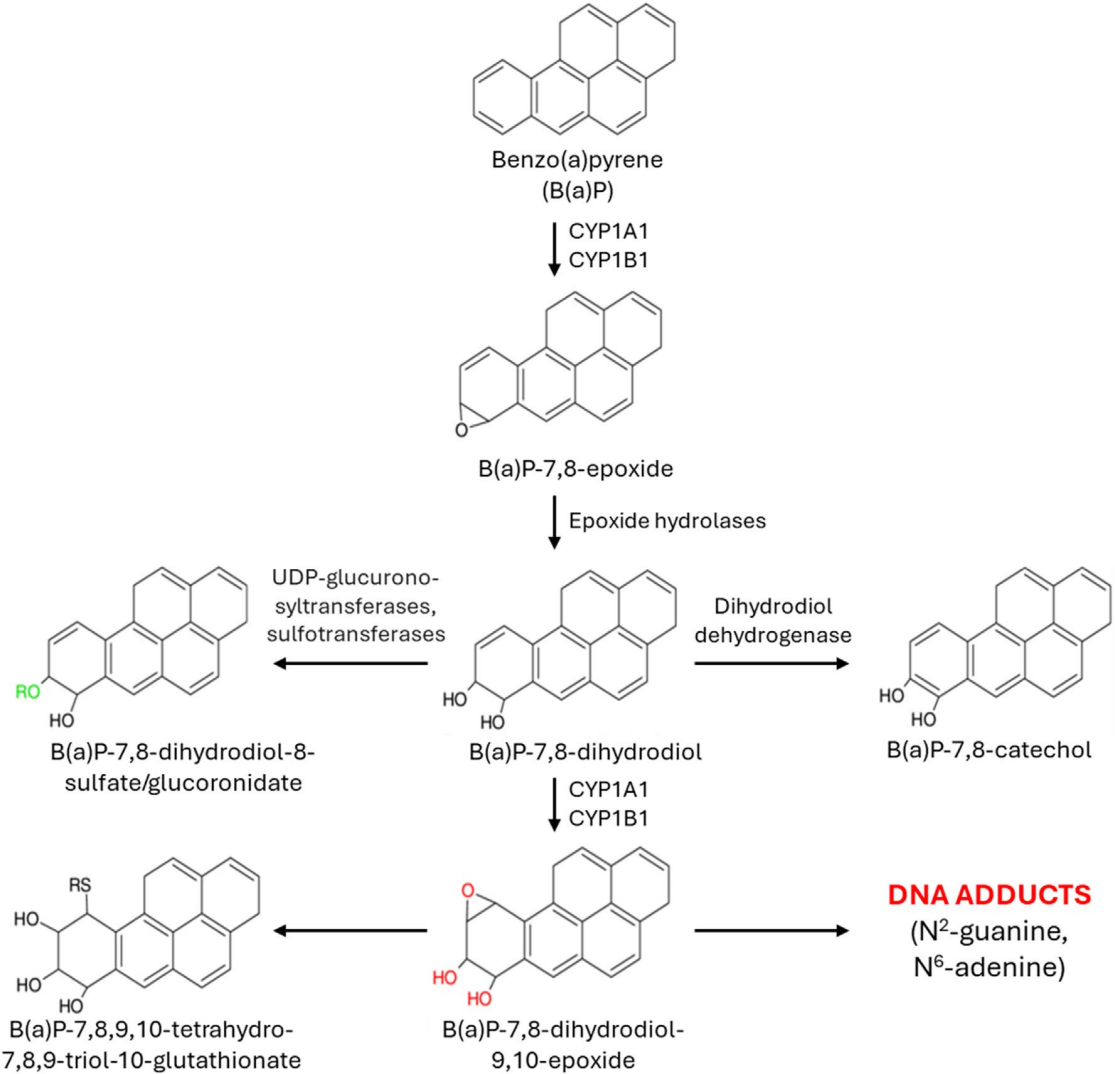
Metabolism of benzo(a)pyrene (B(a)P). Modifications on the ring responsible for increased or decreased reactivity are shown in red and green colors, respectively. CYP, cytochrome P450.

Demonstration of target tissue exposure is critical in validating a NAM that can be used as a follow‐up to an in vitro assay. Therefore, the goal of this study was to verify that in CEGA tested chemicals can reach the fetal chicken liver (target organ) following administration into the air sac of the fertilized egg at sufficient levels to produce genotoxic effect(s), and to form sufficient amounts of reactive metabolite(s) from a parent compound upon reaching the target tissue. For this analysis, a polycyclic aromatic hydrocarbon (PAH), benzo(a)pyrene (B(a)P) was chosen. Many of the chemicals that belong to the PAH group are genotoxic carcinogens (Urwin et al. 2024). Carcinogenic activity of B(a)P involves activation of the Aryl hydrocarbon receptor (AhR), which in turn binds to AhR nuclear translocator and induces the expression of genes involved in B(a)P bioactivation and detoxification. These genes are the cytochrome P450 (CYP) genes CYP1A1, CYP1A2, CYP1B1, as well as glutathione transferase (GST) and Uridine diphosphate (UDP)‐glucuronosyltransferase (UGT‐1). In order to exhibit genotoxicity, B(a)P requires oxidation by phase I CYP1A1 into B(a)P‐7,8‐epoxide, which through hydration by microsomal epoxide hydrolase is metabolized to B(a)P‐7,8‐dihydrodiol (BPD) (Figure 1). BPD is then metabolized to B(a)P‐7,8‐dihydrodiol‐9,10‐epoxide (BPDE) by a second CYP reaction (Kim et al. 2022; Decker et al. 2009). BPDE contains an epoxide ring that is highly reactive with DNA in a time‐dependent manner. In vitro, B(a)P consistently produced negative outcomes in mutagenicity and clastogenicity studies in the absence of metabolic activation, only demonstrating positive outcomes in the presence of exogenous S9 fraction (EPA 2017). In contrast, in CEGA, B(a)P formed DNA adducts and produced DNA strand breaks in the livers of chicken embryo‐fetuses (Williams et al. 2014).

## 2 Materials and Methods

### 2.1 Chemicals

Solutol HS15 (Kolliphor HS15) (CAS: 70142‐34‐6), obtained from Sigma‐Aldrich (St Louis, MO, USA) prepared as a 20% aqueous solution (20% HS15) was used as the vehicle for B(a)P. Deionized water was used as a vehicle for the acridine orange study. B(a)P (CAS: 50‐32‐8; ≥ 96% pure) purchased from Sigma‐Aldrich (St Louis, MO, USA) was used in the metabolomics as well as gene expression studies. Acridine orange (CAS: 10127‐02‐3; pure, ≥ 55% dye content), purchased from Acros Organics (Bridgewater, NJ, USA), was used for the two photon microscopy study.

### 2.2 Egg Handling

The protocol of the study is described in detail in Williams et al. (2014) and Thakkar et al. (2024). Briefly, fertilized eggs (SPF Premium) of white leghorn chicken ( *Gallus gallus* ) were purchased from Charles River Laboratories (North Franklin, CT). Eggs were weighed, numbered, and randomly divided into control and dosed groups (at least 10 eggs per group). On day 0 incubation day, eggs were placed in automatic egg turners and incubated in GQF Manufacturing Company Hova Bator Model 2362N Styrofoam incubators (Murray McMurray Hatchery, Webster City, IA, USA) at 37°C ± 0.5°C and 60% ± 5% humidity. Viability was assessed by transillumination on incubation day 8, and eggs that did not develop were discarded. Separate incubators were used for control and dosed eggs to avoid any possible cross contamination. Doses of compounds were selected based on available acute toxicity data (oral LD50 in rodents, extrapolated on ~60 g egg). For imaging studies, acridine orange was administered at 10 μg/egg. For analysis of metabolites and genomic changes, B(a)P was injected at 250 μg/egg. Test compounds and respective vehicles were administered in a total volume of 0.15 mL/egg via three daily injections into the air sac on incubation days 9 through 11. For metabolite and gene expression analyses, a group of naïve (non‐dosed) eggs that did not receive any injections was also included.

The eggs were terminated 2–3 h after the last injection. The eggshells were opened, the fetuses removed, and decapitated. Fetal weights, including the head, were recorded after removal of the surrounding excess yolk. Viability percentage was calculated based on the ratio of embryo‐fetuses alive upon termination to the total number of embryo‐fetuses in the group. The abdominal cavity was opened, and the livers were removed, weighed, and processed for further analyses.

### 2.3 Two‐Photon Microscopy

#### 2.3.1 Instrument Setup

Two‐photon imaging of tissue samples was performed using the Leica Stellaris 8 DIVE system (Leica Microsystems, Wetzlar, Germany). The microscope is equipped with a mode‐locked titanium: sapphire laser for excitation, capable of delivering femtosecond pulses at the desired wavelength. The laser power and wavelength were optimized based on the fluorophores for acridine orange (460/650). The microscope was configured for both two‐photon excitation and detection, allowing for deep tissue imaging with high spatial resolution.

#### 2.3.2 Sample Mounting

Prior to imaging, tissue samples were mounted onto a slide and a drop of water was added with a coverslip mounted on top. Care was taken to ensure that the sample was securely positioned and oriented for optimal imaging.

#### 2.3.3 Imaging Parameters

The imaging parameters, including laser power, wavelength, scanning speed, and image resolution, were carefully optimized. Laser power was adjusted to achieve sufficient signal intensity while minimizing photobleaching and phototoxicity. The scanning speed was optimized to balance imaging speed with signal‐to‐noise ratio and resolution requirements. Z‐stack imaging was performed to capture three‐dimensional information about the tissue structure, with the step size adjusted based on the desired axial resolution.

#### 2.3.4 Image Acquisition

Two‐photon imaging was performed using optimized parameters, with image acquisition conducted in both *x*, *y*, and *z* dimensions. Z‐stack images were acquired by scanning through the tissue volume at consecutive focal planes. Care was taken to minimize exposure to laser light and phototoxic effects on the sample during image acquisition.

### 2.4 LC‐HRMS

Frozen liver samples were sent to Frontage Laboratories (Exton, PA) for the analysis using LC‐HRMS with Xcalibur and Freestyle Compound Discoverer software.

#### 2.4.1 Sample Preparation

Liver samples were weighed in the nonskirted homogenizing tube containing 0.5 mm zirconium and mixed with 9‐fold of IPA/H_2_O = 70:30 (weight:volume = 1 g:9 mL) followed by 45 s homogenization at 4000 cycles per minute. The homogenized liver samples were volume proportional pooled into three separate mixtures by treatment (untreated, solvent treated, BP treated). 100 μL of pooled sample was mixed with 200 μL organic solvent (ACN with 0.1 μg/mL ISD). Then vortexed and centrifuged for 5 min at 13,000 rpm. Take 250 μL of supernatant and dry it down to 100 μL under N2 prior to LC/HRMS.

#### 2.4.2 Instrumentation

The analytical instrumentation utilized in this study consisted of a Thermo Scientific Vanquish Ultra‐performance liquid chromatography (UPLC) system equipped with multiple units identified by serial numbers: 8315629, 8315641, 8315545, and 6504418. Coupled to the UPLC system was a Thermo Scientific Q Exactive mass spectrometer identified by the serial number 10374 L.

#### 2.4.3 UPLC Conditions

For chromatographic separation, a mobile phase comprising 0.1% formic acid in water (Mobile Phase A) and 0.1% formic acid in acetonitrile (Mobile Phase B) was employed. The separation was achieved on a Phenomenex Kinetex BP column (2.6 × 100 mm) using a gradient elution program with varying percentages of Mobile Phase B over time: 5% at 0 min, 5% at 1 min, 75% at 7 min, 95% at 10 min, maintaining 95% until 12 min, returning to 5% at 12.5 min, and equilibrating at 5% until 15 min. The flow rate was set at 0.4 mL/min, and injection volumes ranged from 2 to 10 μL.

#### 2.4.4 Mass Spec Conditions

The mass spectrometer was operated in positive ionization mode with a spray voltage of 3.50 kV. Additional parameters included an S‐lens RF level of 55, probe heater temperature set at 375°C, and capillary temperature maintained at 325°C. The sheath gas flow rate was set to 45 units, with auxiliary gas at 15 units and sweep gas at 1 unit. Mass spectra were acquired over a range of m/z 150–850 with a full MS resolution of 35,000 and an automatic gain control (AGC) target of 3e6. MS/MS experiments were conducted at a resolution of 17,500, with an AGC target of 1e5, using collision energies (CE) of 30, 40, and 55.

#### 2.4.5 Reagents

Reagents used in the analysis included Fisher Optima LC/MS grade solvents: water, acetonitrile, methanol, and formic acid. These reagents were chosen to ensure high purity and compatibility with the analytical instrumentation employed in this study.

### 2.5 RNA Sequencing

RNA extraction and sequencing from the liver samples was performed at Azenta Life Sciences (South Plainfield, NJ).

#### 2.5.1 RNA Extraction

Total RNA was extracted using the Qiagen Rneasy Plus Mini kit following the manufacturer's instructions (Qiagen, Hilden, Germany).

#### 2.5.2 Library Preparation With PolyA Selection and Illumina Sequencing

Quantification of RNA samples was done using the Qubit 2.0 Fluorometer (Life Technologies, Carlsbad, CA, USA) and RNA integrity assessment was done using the Agilent TapeStation 4200 (Agilent Technologies, Palo Alto, CA, USA). Prior to library preparation, the ERCC RNA Spike‐In Mix (Cat: 4456740) from ThermoFisher Scientific was added to normalized total RNA following the manufacturer's protocol. NEBNext Ultra II RNA Library Prep Kit was used to prepare RNA sequencing libraries (NEB, Ipswich, MA, USA). Enrichment of mRNAs with Oligod(T) beads for a brief time. In the next step at 94°C enriched mRNAs were fragmented for 15 min. Both first and second strand cDNA were synthesized. The cDNA fragments were then end‐repaired and adenylated at the 3′ ends. Universal adapters were ligated to the cDNA fragments, followed by the addition of indexes and library enrichment through PCR with a limited number of cycles. The validation of the sequencing library was done on Agilent TapeStation (Agilent Technologies, Palo Alto, CA, USA), and quantification was done by using Qubit 2.0 Fluorometer (Invitrogen, Carlsbad, CA) along with quantitative PCR (KAPA Biosystems, Wilmington, MA, USA). On a flowcell the sequencing libraries were clustered. The flowcell was loaded on the Illumina NovaSeq instrument post clustering. Using a 2x150bp Paired End (PE) configuration, the samples were sequenced as a next step. Control software was used to do image analysis and base calling. Raw sequence data (bcl files) generated by the sequencer were converted into fastq files and de‐multiplexed using Illumina's bcl2fastq 2.17 software. One mismatch was allowed for index sequence identification.

#### 2.5.3 Data Analysis

After investigating the quality of the raw data, Trimmomatic v.0.36 was used to trim sequence reads remove possible adapter sequences and poor‐quality nucleotides. The trimmed reads were aligned to the chicken ( *gallus gallus* ) reference genome available on ENSEMBL using the STAR aligner v.2.5.2b, resulting in the generation of BAM files. Unique gene hit counts were calculated using featureCounts from the Subread package v.1.5.2, counting only unique reads that fell within exon regions. The gene hit counts table was then used for downstream differential expression analysis. DESeq2 was employed to compare gene expression between the sample groups, using the Wald test to generate *p*‐values and log2 fold changes. Genes with adjusted *p*‐values < 0.05 and absolute log2 fold changes > 1 were identified as differentially expressed for each comparison. Gene ontology analysis was performed on statistically significant genes using the GeneSCF software, clustering the genes based on their biological processes and determining their statistical significance using the human GO list.

The gene code was converted to gene symbol using Biotools.fr (https://www.biotools.fr/mouse/ensembl_symbol_converter, frEnsembl Gene Database, n.d.). STRING v. 12.0 and Cytoscape v. 3.1 databases were used for gene mapping, functional enrichment analysis, and network visualization.

## 3 Results

### 3.1 Target Tissue Exposure

**FIGURE 2:**
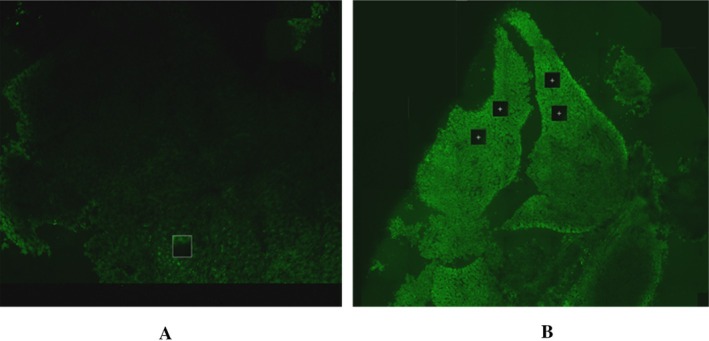
Whole liver imaging using two‐photon excitation microscopy. Images were taken at the same settings for control (A) and dose (B) groups at 500/526 excitation emission spectra using the Stellaris8 multiphoton imaging system and at ×20 magnification. (A) Vehicle (deionized water) control group. (B) Acridine orange dosed group (10 μg/egg).

The results from multiphoton imaging are represented in Figure 2. The visual analysis of the fluorescence intensity indicated that liver tissue in the group that received injections with 10 μg/egg of fluorescent dye, acridine orange, demonstrated an increase in fluorescent staining (Figure 2B) compared to the control group, which received injections with deionized water only (Figure 2A). This confirms sufficient liver uptake of acridine orange, which has reached the target tissue following its administration into the air sac.

### 3.2 B(a)P Metabolism in CEGA

The viability in the group dosed with B(a)P at a total dose of 250 μg/egg was 100%. The viability in the vehicle control group was also 100%.

**FIGURE 3:**
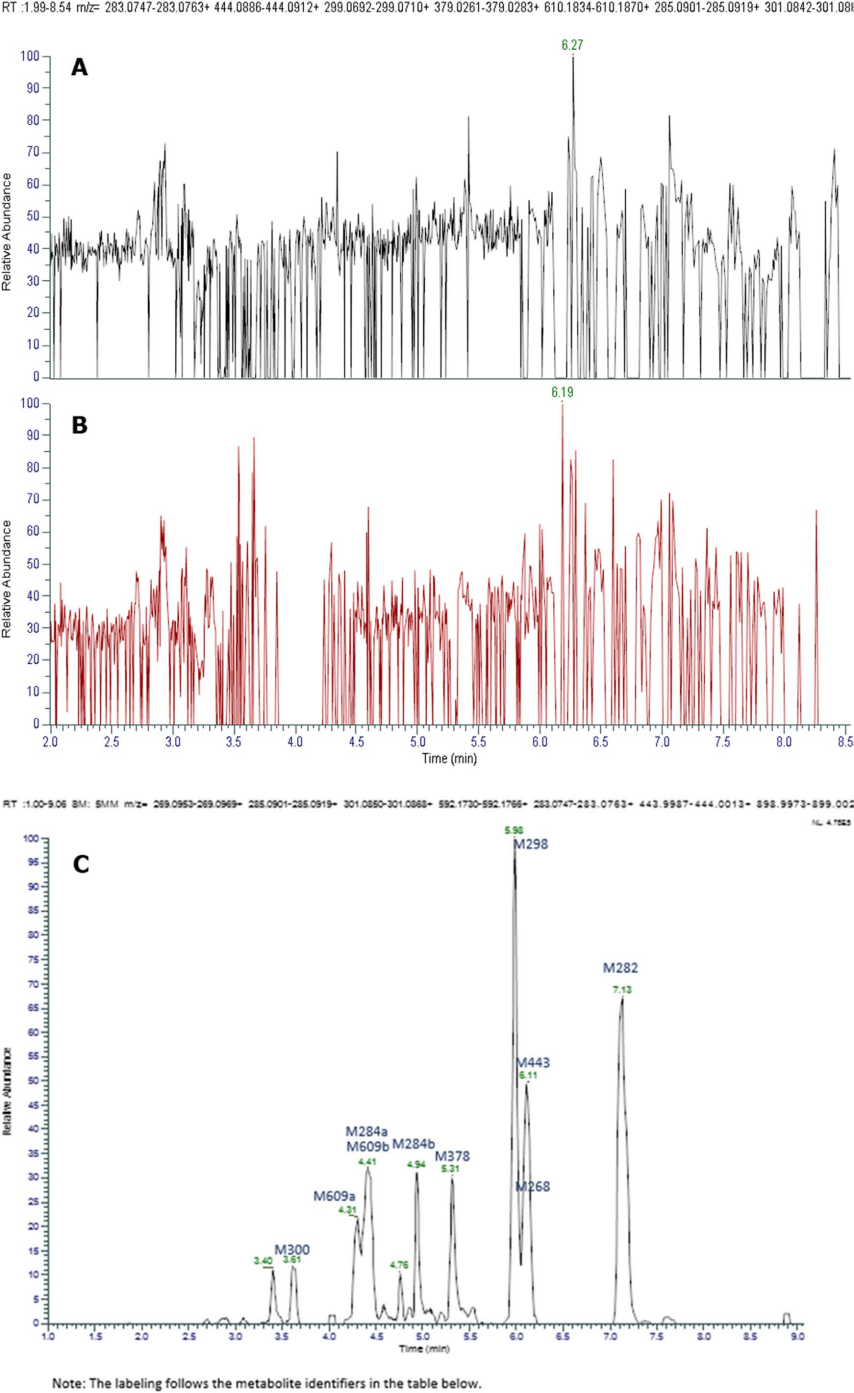
Analysis of metabolites formed over the time in the embryo‐fetal chicken liver from the untreated (A), solvent control (B), and benzo(a)pyrene B(a)P‐dosed group (C). The *Y*‐axis represents measured relative absorbance and the *X*‐axis represents retention time in minutes. Each peak (m/z ratio) represent molecules with different mass corresponding to a distinct metabolite, while the area under each peak represents the amount of each metabolite formed.

**FIGURE 4:**
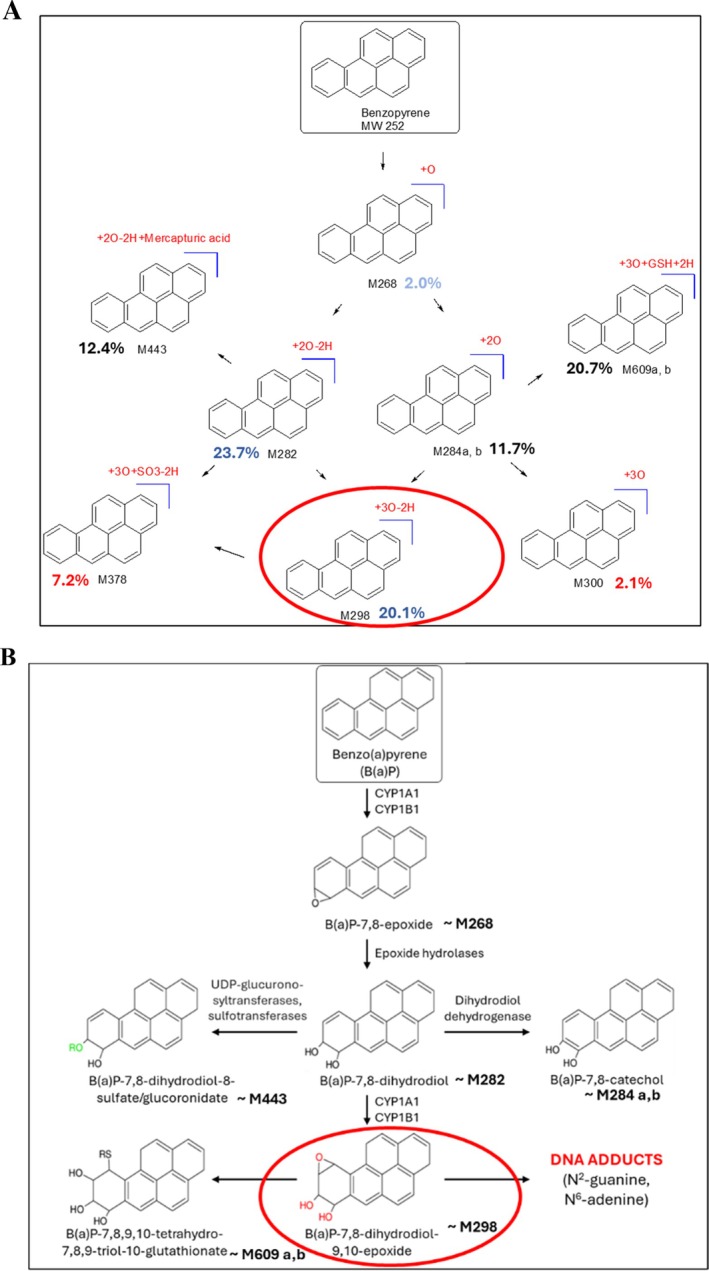
Comparison of benzo(a)pyrene (B(a)P) metabolism in the chicken embryo‐fetus (A) and rodents (B). In panel A, the modifications on B(a)P ring detected in CEGA are listed in red, and their respective amounts in percentages are also provided. In panel B, B(a)P metabolites assumed to be similar to those detected in CEGA are marked with a corresponding metabolite identification number. The metabolite circled in red is the DNA‐reactive metabolite capable of covalent DNA binding. (A) Proposed B(a)P metabolism in CEGA. (B) Established B(a)P metabolism in rodents.

The results of the metabolism study following B(a)P exposure are shown in Figure 3. No relevant peaks were observed in either naïve (untreated) (Figure 3A) or solvent control (Figure 3B) groups, indicating that no metabolites were formed in these groups. In contrast, in the livers of chicken embryo‐fetuses that received injections with B(a)P, several peaks representing distinct metabolites with transformations on B(a)P ring were formed at sufficient levels (Figure 3C). The quantification and the type of each transformation are listed in Table 1. These transformations were compared to the established metabolism of B(a)P in other species (Figure 4). Based on the suggested metabolite structures, there seems to be similarity in the metabolites formed between chicken and rodent livers (Figure 4). Specifically, the most abundant metabolites in the chicken fetal livers were M282 and M298 (Table 1). These metabolites are assumed to correspond to B(a)P‐7,8‐dihydridiol and DNA‐reactive B(a)P‐7,8‐dihydridiol‐9,10‐epoxide formed in rodents (Figure 4).

**TABLE 1:** Quantification of metabolites detected in the chicken embryo–fetal livers following administration of benzo(a)pyrene (B(a)P).

Identifier	RT (min)	Observed (m/z)	Accuracy (ppm)	Biotransformation of B(a)P	% MS
M300	3.63	301.0851	−2.7	+3O	2.1
M609a	4.28	610.1852	−0.3	+3O + GSH + 2H	14.7
M284a	4.41	285.0910	0.0	+2O	6.5
M609b	4.44	610.1852	−0.3	+3O + GSH + 2H	6.0
M284b	4.93	285.0910	0.0	+2O	5.2
M378	5.32	379.0272	0.3	+3O‐2H + SO_3_	7.2
M298	6.00	299.0701	−2.3	+3O‐2H	20.1
M268	6.02	269.0956	−1.9	+O	2.0
M443	6.11	444.0899	−0.2	+2O‐2H + Mercapturic acid	12.4
M282	7.12	283.0755	−1.4	+2O‐2H	23.7

*Note:* % was calculated by MS response.

Abbreviations: Gluc, glucuronide; GSH, glutathione; RT, retention time.

### 3.3 Differential Gene Expression in Response to B(a)P

**FIGURE 5:**
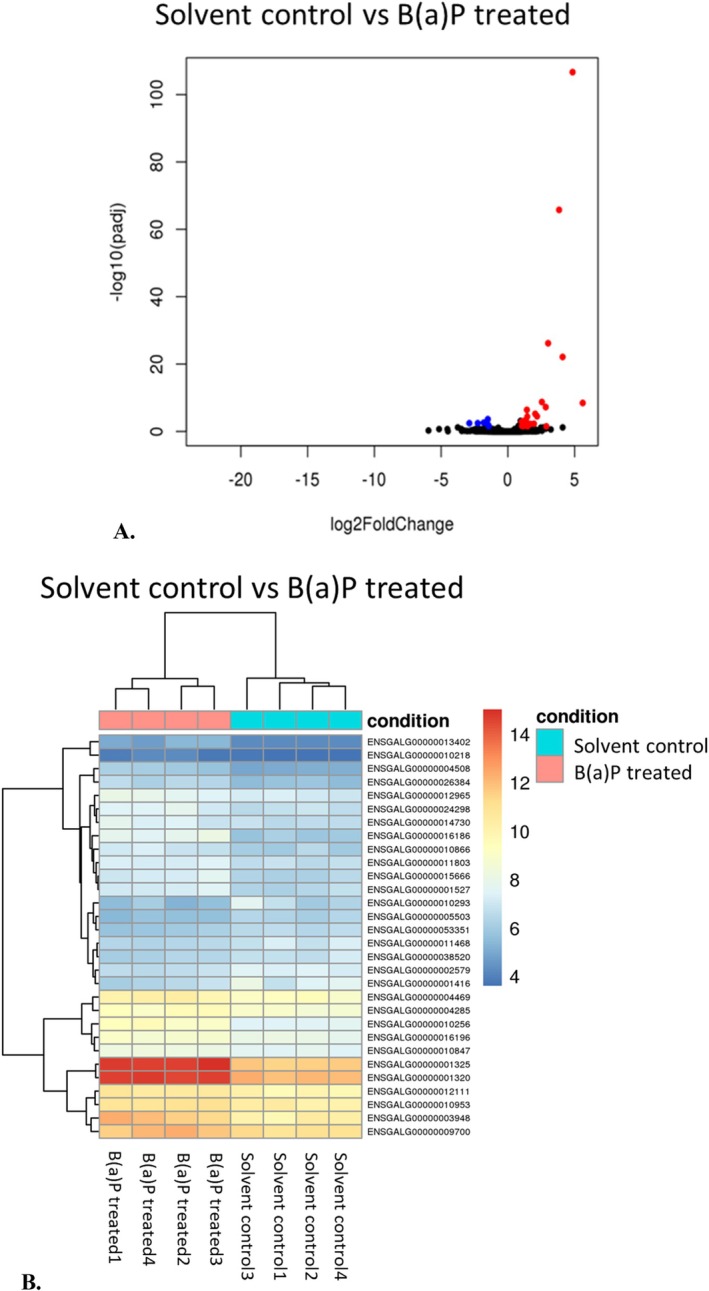
Volcano plot (A) showing differentially expressed genes. *X*‐Axis represents log2fold change and *Y*‐axis represents −log10 (padj). Heatmap (B) showing top 30 significant differentially expressed genes. Benzo(a)pyrene was administered at 250 μg/egg.

The heatmap (Figure 5) demonstrates expression profiles of the top 30 differentially expressed genes which were sorted by their adjusted *p*‐values to identify up‐or down‐regulated genes across control and dose groups. Ten genes which were significantly deregulated by B(a)P in the livers of chicken embryo‐fetuses (were identified Table 2). Out of these, eight genes were upregulated and two were downregulated in the B(a)P dose group when compared to the control group.

**TABLE 2:** List of the most abundantly up‐ and down‐regulated genes by benzo(a)pyrene (B(a)P) in the livers of chicken embryo‐fetuses.

Gene ID	Gene symbol	Acronym	Function	Log2fold change	*p*
ENSGALG00000013402	ArhRR	Aryl‐hydrocarbon receptor repressor	CYP1A1 activity	5.606	3.65E−09
ENSGALG00000001325	CYP1A1		CYP1A1 activity	4.847	2.14E−107
ENSGALG00000016186	PDE9A		cGMP activity	4.099	7.96E−23
ENSGALG00000001320	CYP1A2		CYP1A2 activity	3.836	1.71E−66
ENSGALG00000010256	TIPARP	TCDD inducible poly (ADP‐ribose) polymerase	CYP1A1 activity	3.014	6.71E−27
ENSGALG00000004508	EYA2	EYA transcriptional coactivator and phosphatase 2	H2Ax; DNA repair activation	2.835	6.08E−08
ENSGALG00000003948	ALAS1	5′‐aminolevulinate synthase 1	Catalyzes the rate‐limiting step in heme (iron‐protoporphyrin) biosynthesis	2.546	1.87E−09
ENSGALG00000026384	PCSK4	Proprotein convertase subtilisin/kexin type 4	Subtilisin‐like proprotein convertase family	2.184	3.27E−05
ENSGALG00000001416	ADRA1B	adrenoceptor alpha 1B	Alpha‐1‐adrenergic receptors (alpha‐1‐ARs) are members of the G protein‐coupled receptor superfamily	−2.259	0.00376281
ENSGALG00000010293	RBP	Riboflavin transport		−2.887	6.23 E−06

The genes that had direct and/or indirect effect on CYP1A1 activity were ENSGALG00000013402; ENSGALG00000001325; ENSGALG00000001320; and ENSGALG00000010256. All these four genes were upregulated. ENSGALG00000016186 which affected cyclic GMP activity was also upregulated. Other three genes which were upregulated were ENSGALG00000004508; ENSGALG00000003948 and ENSGALG00000026384 which are involved in DNA repair activity; heme formation and proprotein conversion, respectively. The two downregulated genes namely, ENSGALG00000001416 and ENSGALG00000010293 play an important role in alpha‐1 adrenergic receptor activity and riboflavin transport activity, respectively.

**FIGURE 6:**
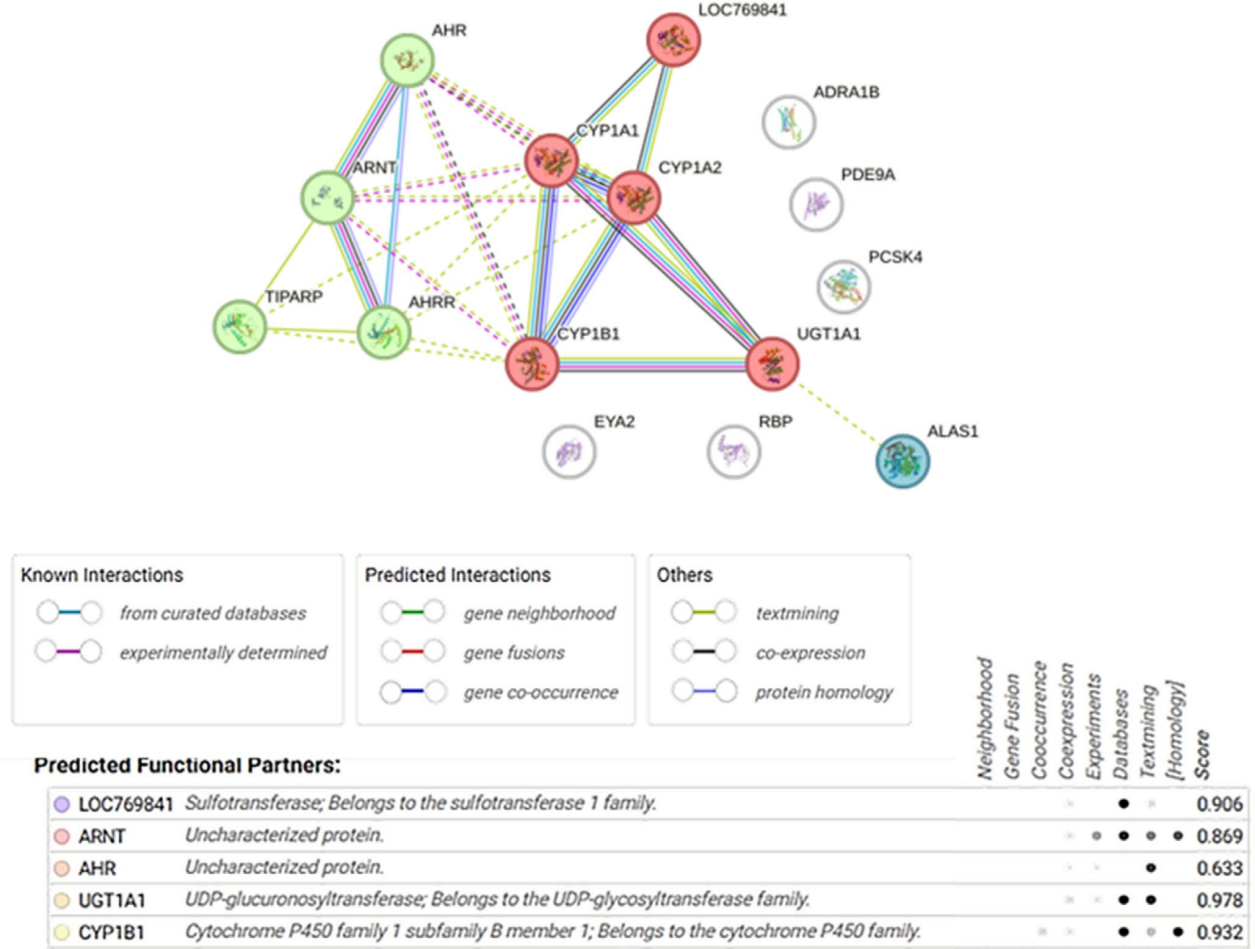
Protein–protein interactions among the identified significantly expressed genes. Figure was created using String platform (https://string‐db.org/) with k‐means clustering. Network nodes represent queried proteins. Colored nodes represent the first shell of interactors, white nodes represent second shell of interactors. Nodes are filled in with a known or predicted 3D structure of the protein. Edges represent protein–protein associations.

Functional relationships of significantly deregulated genes and their protein–protein interaction network are shown in Figure 6. The majority of genes participate in shared biological pathways or processes, in particular, xenobiotic metabolic process, aromatase activity, and aryl hydrocarbon receptor complex (Supporting Information Table 1).

**FIGURE 7:**
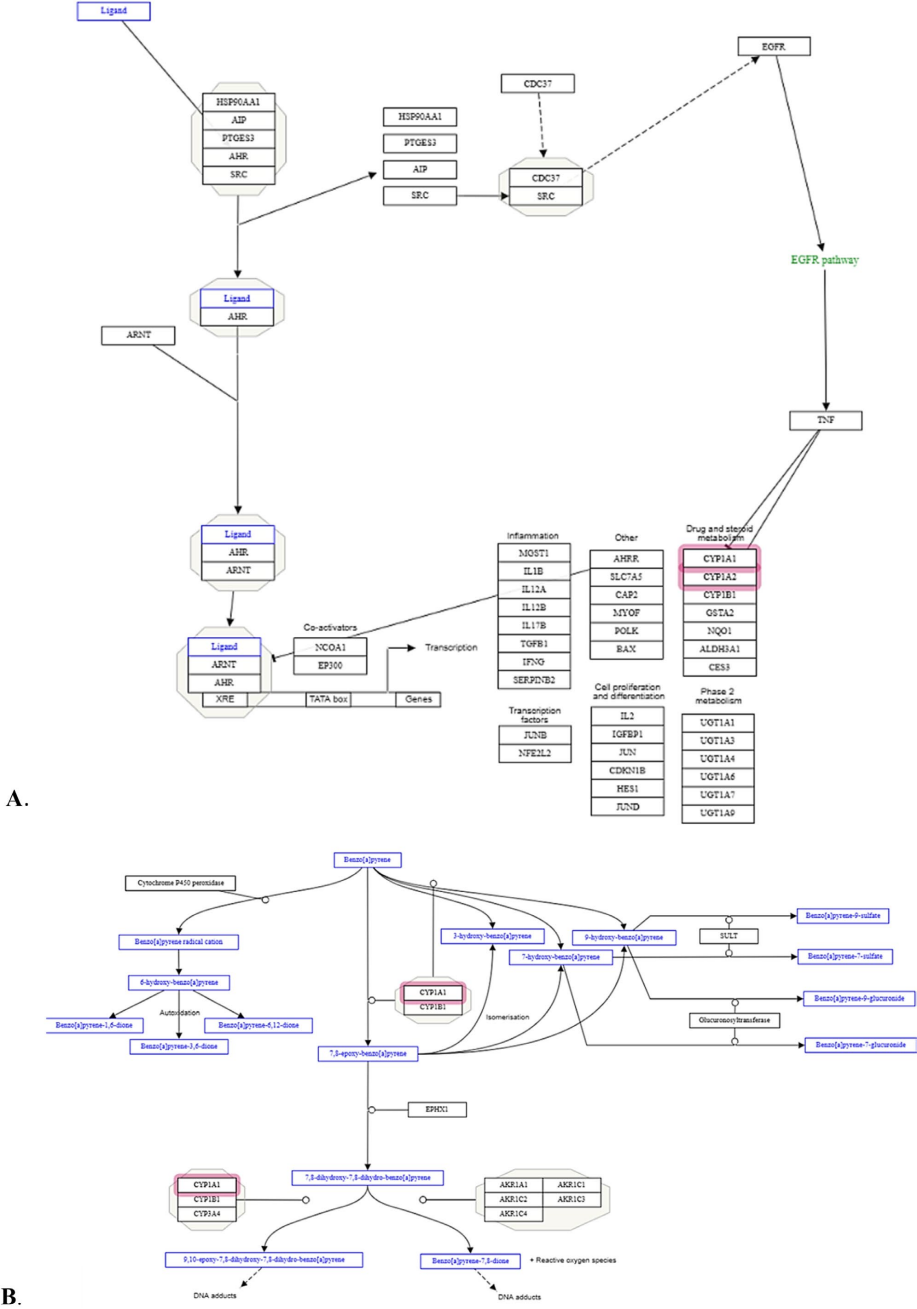
Network visualization for selected enriched pathways. (A) WikiPathway WP2873—Aryl hydrocarbon receptor pathway— *Homo sapiens* . (B) WP696—Benzo(a)pyrene metabolism— *Homo sapiens* . Figure of curated pathways was created using Cytoscape database (https://cytoscape.org/). Query genes are highlighted in red boxes.

Pathway enrichment analysis of human orthologs of identified differentially expressed genes revealed several enriched pathways, notably the arylhydrocarbon receptor pathway (*p* = 0.0023) and B(a)P metabolism pathway (*p* = 0.015) (Figure 7).

These findings were in concordance with a statistically significant increase in the activity of the CYP1A1 enzyme in the chicken embryo‐fetal livers following injections of B(a)P at 125 and 250 μg/egg (Supporting Information Figure 1).

## 4 Discussion

To evaluate the CEGA model as an in vivo NAM, it is critical to assess target tissue exposure. Since the injections in CEGA are conducted into the air space of fertilized eggs, xenobiotics must cross a layer of eggshell membrane in order to reach the target tissue, namely liver. To demonstrate target tissue exposure and sufficient metabolism after reaching target tissue, multiphoton microscopy, metabolite analysis, and genomic studies were conducted.

For multiphoton microscopy, acridine orange was selected as an appropriate fluorescence dye since the molecular weight of acridine orange is in the range of B(a)P, which was used for metabolism and target tissue exposure studies. The results indicated that even though the chemical was injected into the airspace, it penetrated the eggshell membrane and reached the liver (Figure 2), resulting in sufficient liver uptake. It is important to mention that the staining pattern in the dose‐group was not uniform across the whole liver, which might indicate different uptake of the compound by different liver lobes. This aspect requires further investigation.

To study metabolism in CEGA, B(a)P was used as a chemical of choice, since it is known to produce its genotoxic effect post metabolic activation. Moreover, previous studies in CEGA confirmed that B(a)P forms DNA adducts and DNA strand breaks in the livers of chicken embryo‐fetuses (Williams et al. 2014). B(a)P‐7,8 dihydrodiol‐9,10‐epoxide is the reactive metabolite known to covalently bind to DNA, forming adducts and producing adverse effects (Figure 1). Additionally, metabolism of B(a)P has been well documented in animal studies, which allows for comparison between the metabolites formed in the livers of chicken embryo‐fetuses and other species. The characterization of metabolites formed in CEGA following exposure to B(a)P confirmed that the majority of the metabolites observed in the rodents were also detected in chickens (Figure 4). Two additional metabolites were formed in the chicken livers. These were identified as M378 and M300, and formed at 7.2% and 2.1%, respectively. M378 metabolite had an addition of three oxygen molecules (3O), removal of two hydrogen groups (−2H) and addition of one sulfate group (SO_3_), whereas M300 metabolite had three additional oxygen (3O) on B(a)P ring. However, M378 formation is also justified and follows a similar pathway as mentioned in the IARC document (IARC 1983) which states that B(a)P is metabolized initially by the microsomal CYP systems to several arene oxides. Once formed, these arene oxides may rearrange spontaneously to phenols, undergo hydration to the corresponding trans‐dihydrodiols in a reaction catalyzed by microsomal epoxide hydrolase, or react covalently with GSH, either spontaneously or in a reaction catalyzed by cytosolic GST (IARC 1983).

Overall, the findings in the metabolism study confirmed that B(a)P metabolism in CEGA aligns with the established metabolic pathway in rodents (Decker et al. 2009). Another similarity was observed with the animal study of orally administered B(a)P in F344 rats, which found a half‐life of B(a)P in rat liver to be 12 h, suggesting that unmodified/unmetabolized B(a)P is 100% converted to its metabolites 24 h post‐exposure (Ramesh et al. 2001). In CEGA, 100% of administered B(a)P was converted to its relevant metabolites. The toxic precursor, (B(a)P‐7,8‐dihydridiol), was formed at 23.1% 48‐h post treatment in embryo‐fetal chicken livers. In rat liver, however, B(a)P‐7,8‐dihydridiol was only present at 10% 48‐h post administration with its peak liver concentration at ~30% 24‐h post treatment after a single dose of 100 mg/kg bw of B(a)P (Ramesh et al. 2001). Formation of the reactive metabolite, B(a)P‐7,8 dihydrodiol‐9,10‐epoxide, which in CEGA was formed at 20%, is likely to be responsible for the formation of DNA adducts observed in the embryo‐fetal chicken livers (Williams et al. 2014). The difference in the amount of metabolite formed in CEGA and the rodent study mentioned above may also be due to the single oral administration in Ramesh et al. (2001) study as compared to three different doses in CEGA studies.

The analyses of the differential gene expression in the embryo‐fetal chicken livers following exposure to B(a)P also confirmed that the compound upregulated the expression of genes responsible for its bioactivation (Table 2, Supporting Information Table 1, Figure 7). Specifically, out of 10 significantly deregulated genes, three were involved in the activity of CYP1A1 and CYP1A2 isoenzymes. Other identified genes were also involved in the activity of DNA strand break‐mediated gene H2AX and DNA repair‐mediated gene RNA polymerase II. These results support the conclusion that B(a)P at a total dose of 250 μg/egg in the embryo‐fetal chicken livers upregulates the expressions of CYP1A genes, which affect the activity of CYP1A1 (Supporting Information Figure 1), leading to the formation of the reactive metabolite BPDE, resulting in DNA damage and activating DNA repair mechanisms.

In addition to the upregulation of genes involved in CYP1A1 activity, an increase was also observed in the expression of genes which regulate CYP1A1 by negative feedback loop. Specifically, the upregulation of AhR repressor and TCDD Inducible Poly (ADP‐Ribose) Polymerase (TiPARP) genes (Table 2) can negatively regulate CYP1A1 activity. This also adds to the fact that with B(a)P treatment, there was a significant increase in CYP1A1 activity, which is a critical enzyme for metabolizing B(a)P leading to a toxic metabolite, but genes regulating CYP1A1 expression by negative feedback mechanism were also present in chicken livers.

Similarities between the expressions of CYP1A1 and CYPA2 genes in the embryofetal chicken livers following dosing with B(a)P were observed with the published data in rodents and human cells. For example, expressions of CYP1A1 and CYP1A2 genes in the livers of Wistar rats that received B(a)P at a single dose of 150 mg/kg bw by oral gavage was significantly increased by 2990 and 27.7 folds, respectively (Dračínská et al. 2021). In the study with human hepatocellular carcinoma cell line (HepG2) which was incubated with 2 μM of B(a)P, CYP1A1 showed a 93‐fold and 79‐fold increase in expression on microarray 12‐ and 24‐h post‐dosing, respectively, whereas RNA‐seq demonstrated a 199‐fold (at 12 h) and 214‐fold (at 24 h) increases in CYP1A1 expression (Van Delft et al. 2010). In the human tissue organoid cultures, differentiated liver had significantly higher (24‐fold) CYP1A1 levels compared to undifferentiated samples at basal level. After exposure to 50 μM of B(a)P, induction of CYP1A1 in differentiated liver organoids was around double compared to that in undifferentiated organoids (~4500‐ and 2000‐fold, respectively), relative to control. At 12.5 μM, a 287‐fold change compared to undifferentiated control was observed only in differentiated organoids. Induction of CYP1A1 was also significant at both concentrations of B(a)P compared to differentiated control (Caipa Garcia et al. 2023). These results fall into the same range compared to what we found for the CEGA, which demonstrated a fold change of 1024 folds for CYP1A1 activity combined (ENSGALG00000013402 and ENSGALG00000001325) and 16 folds for CYP1A2 activity (Table 2).

While the results of the current study overall support the conclusion that CEGA is capable of metabolism and target tissue uptake of genotoxic carcinogens, the study had some limitations. Firstly, the number of chemicals tested in this study was limited. The expansion of metabolism studies in CEGA to other chemical classes which require different bioactivation pathways would significantly strengthen the utility of the model and will further elucidate an ability of embryo‐fetal livers to metabolize chemicals. Target tissue exposure studies can be extended to investigate differences in the uptake of chemicals by different liver lobules, which might elucidate differences in metabolic capabilities within the liver. Moreover, inter‐ and intra‐laboratory validation of CEGA will strengthen its validity and utility as a potential NAM to evaluate the genotoxic potential of chemicals.

## 5 Conclusion

The findings in the current study in CEGA using a known genotoxic chemical that is activated by metabolic transformation demonstrate sufficient liver uptake, metabolism, and gene expression modulations that resemble those of rodents. This data confirms the utility of the model as a NAM for the assessment of the genotoxic potential of chemicals. CEGA can specifically be used as a follow‐up assay for the genotoxicity assessment of chemicals that were genotoxic in vitro. In particular, this model can be useful for the assessment of chemicals that require metabolic bioactivation and/or sufficient systemic exposure.

## Conflicts of Interest

The authors declare no conflicts of interest.

## Supporting information


**Fig. S1.** **Activity of CYP1A1 enzyme in the embryo‐fetal chicken livers following exposure to benzo(a)pyrene (B(a)P)**. Total dose represents cumulative dose, administered in 3 daily injections on incubation days 9 through 11. The assay was conducted using CYP1A1 detection kit (Catalog#V8751) from Promega Corporation (Madison, WI, USA) according to the manufacturer's protocol. luciferin‐CEE was used as a substrate
Supplemental Table 1. **Functional enrichments of queried significantly deregulated genes**.
