# Supplementary material for: The Chicken Egg Genotoxicity Assay (CEGA): Assessing Target Tissue Exposure and Metabolism in the Embryo‐Fetal Chicken Livers
Source: Environ Mol Mutagen. 2025 May 12;66(5):243–57. doi: 10.1002/em.70015 (PMC12235068; doi:10.1002/em.70015)
Supplement: Supplementary file 1 — Fig. S1. Activity of CYP1A1 enzyme in the embryo‐fetal chicken livers following exposure to benzo(a)pyrene (B(a)P). Total dose represents cumulative dose, administered in 3 daily injections on incubation days 9 through 11. The assay was conducted using CYP1A1 detection kit (Catalog#V8751) from Promega Corporation (Madison, WI, USA) according to the manufacturer's protocol. luciferin‐CEE was used as a substrate Supplemental Table 1. Functional enrichments of queried significantly deregulated genes. [file EM-66-243-s001.docx]

# SUPPLEMENTARY MATERIALS

| 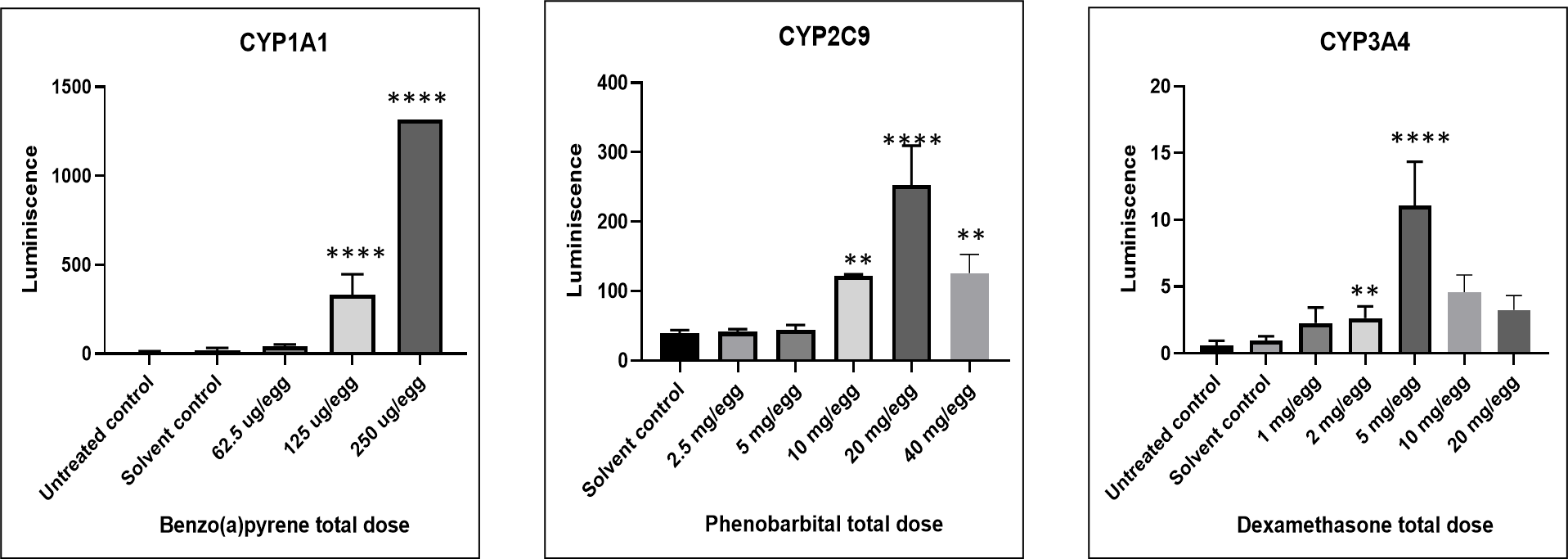 |
| --- |
| Suppl. Fig. 1. **Activity of CYP1A1 enzyme in the embryo-fetal chicken livers following exposure to benzo(a)pyrene (B(a)P).** Total dose represents cumulative dose, administered in 3 daily injections on incubation days 9 through 11**.** The assay was conducted using CYP1A1 detection kit (Catalog#V8751) from Promega Corporation (Madison, WI, USA) according to the manufacturer’s protocol. luciferin-CEE was used as a substrate |

Supplemental Table 1. **Functional enrichments of queried significantly deregulated genes**

| **Term ID** | **Term description** | **Observed gene count** | **Background gene count** | **Strength** | **Signal** | **False discovery rate** | **Matching proteins in the network** |
| --- | --- | --- | --- | --- | --- | --- | --- |
| Biological Process | | | | | | | |
| GO:0006805 | Xenobiotic metabolic process | 5 | 59 | 2.01 | 2.23 | 1.22E-05 | CYP1A2, CYP1A1, AHR, CYP1B1, AHRR |
| Molecular Function | | | | | | | |
| GO:0070330 | Aromatase activity | 3 | 13 | 2.44 | 1.58 | 0.00069 | CYP1A2, CYP1A1, CYP1B1 |
| Cellular Component | | | | | | | |
| GO:0034751 | Aryl hydrocarbon receptor complex | 3 | 9 | 2.6 | 1.95 | 0.00013 | AHR, ARNT, AHRR |
| Local Network Cluster (STRING) | | | | | | | |
| CL:3288 | Steroid hormone biosynthesis | 4 | 12 | 2.6 | 2.82 | 2.08E-06 | CYP1A2, UGT1A1, CYP1A1, LOC769841 |
| CL:24010 | Mixed, incl. Ubiquitin family, and WWE domain, subgroup | 2 | 5 | 2.68 | 1.12 | 0.0064 | TIPARP, AHRR |
| CL:3291 | Atorvastatin ADME, and Cytochrome P450, E-class, group I, CYP1 | 2 | 5 | 2.68 | 1.12 | 0.0064 | UGT1A1 ,CYP1A1 |
| CL:3302 | Mixed, incl. Response to mycotoxin, and Cytochrome P450, E-class, group I, CYP2D-like | 2 | 5 | 2.68 | 1.12 | 0.0064 | CYP1A2, LOC769841 |
| CL:19140 | Motif C-terminal to PAS motifs (likely to contribute to PAS structural domain), and Peptidyl-proline 4-dioxygenase activity | 2 | 15 | 2.2 | 0.78 | 0.0276 | AHR, ARNT |
| KEGG Pathways | | | | | | | |
| gga00140 | Steroid hormone biosynthesis | 5 | 30 | 2.3 | 3.72 | 9.57E-09 | CYP1A2, UGT1A1, CYP1A1, CYP1B1, LOC769841 |
| gga00980 | Metabolism of xenobiotics by cytochrome P450 | 4 | 29 | 2.22 | 2.81 | 1.01E-06 | CYP1A2, UGT1A1, CYP1A1, CYP1B1 |
| gga00380 | Tryptophan metabolism | 3 | 36 | 2 | 1.7 | 0.00023 | CYP1A2, CYP1A1, CYP1B1 |
| gga00830 | Retinol metabolism | 3 | 36 | 2 | 1.7 | 0.00023 | CYP1A2, UGT1A1, CYP1A1 |
| gga00860 | Porphyrin and chlorophyll metabolism | 2 | 21 | 2.06 | 1.11 | 0.0054 | UGT1A1, ALAS1 |
| Reactome Pathways | | | | | | | |
| GGA-211859 | Biological oxidations | 6 | 126 | 1.76 | 2.39 | 9.53E-07 | UGT1A1, AHR, CYP1B1, LOC769841, ARNT, AHRR |
| GGA-211976 | Endogenous sterols | 4 | 18 | 2.43 | 2.8 | 1.74E-06 | AHR, CYP1B1, ARNT, AHRR |
| GGA-8937144 | Aryl hydrocarbon receptor signalling | 3 | 5 | 2.86 | 2.53 | 1.02E-05 | AHR, ARNT, AHRR |
| GGA-211981 | Xenobiotics | 3 | 11 | 2.51 | 2.13 | 5.21E-05 | AHR, ARNT, AHRR |
| GGA-9753281 | Paracetamol ADME | 2 | 18 | 2.12 | 0.78 | 0.0276 | UGT1A1, LOC769841 |
| Subcellular Localization | | | | | | | |
| GOCC:0034751 | Aryl hydrocarbon receptor complex | 3 | 9 | 2.6 | 1.91 | 0.00016 | AHR, CYP1B1, ARNT |
| Protein Domain and Features | | | | | | | |
| PF00067 | Cytochrome P450 | 3 | 47 | 1.88 | 0.85 | 0.0174 | CYP1A2, CYP1A1, CYP1B1 |
| IPR008066 | Cytochrome P450, E-class, group I, CYP1 | 2 | 2 | 3.08 | 1 | 0.0116 | CYP1A2, CYP1A1 |
| SM00091 | PAS domain | 3 | 29 | 2.09 | 1.35 | 0.0017 | AHR, ARNT, AHRR |
| SM00353 | Helix loop helix domain | 3 | 102 | 1.55 | 0.7 | 0.0306 | AHR, ARNT, AHRR |
